# Impact of molecular and clinical variables on survival outcome with immunotherapy for glioblastoma patients: A systematic review and meta‐analysis

**DOI:** 10.1111/cns.13915

**Published:** 2022-07-13

**Authors:** Wentao Hu, Hongyu Liu, Ze Li, Jialin Liu, Ling Chen

**Affiliations:** ^1^ School of Medicine Nankai University Tianjin China; ^2^ Department of Neurosurgery First Medical Center of Chinese PLA General Hospital Beijing China

**Keywords:** glioblastoma multiforme, immunotherapy, meta‐analysis, predictive factor

## Abstract

**Background:**

Given that only a subset of patients with glioblastoma multiforme (GBM) responds to immuno‐oncology, this study aimed to assess the impact of multiple factors on GBM immunotherapy prognosis and investigate the potential predictors.

**Methods:**

A quantitative meta‐analysis was conducted using the random‐effects model. Several potential factors were also reviewed qualitatively.

**Results:**

A total of 39 clinical trials were included after screening 1317 papers. Patients with O6‐methylguanine‐DNA methyltransferase (MGMT) promoter methylation [hazard ratio (HR) for overall survival (OS) = 2.30, *p* < 0.0001; HR for progression‐free survival (PFS) = 2.10, *p* < 0.0001], gross total resection (HR for OS = 0.70, *p* = 0.02; HR for PFS = 0.56, *p* = 0.004), and no baseline steroid use (HR for OS = 0.52, *p* = 0.0002; HR for PFS = 0.61, *p* = 0.02) had a relatively significant favorable OS and PFS following immunotherapy. Patients with a Karnofsky Performance Status score < 80 (HR = 1.73, *p* = 0.0007) and undergoing two prior relapses (HR = 2.08, *p* = 0.003) were associated with worse OS. Age, gender, tumor programmed death‐ligand 1 expression, and history of chemotherapy were not associated with survival outcomes. Notably, immunotherapy significantly improved the OS among patients undergoing two prior recurrences (HR = 0.40, *p* = 0.008) but not among patients in any other subgroups, as opposed to non‐immunotherapy.

**Conclusion:**

Several factors were associated with prognostic outcomes of GBM patients receiving immunotherapy; multiple recurrences might be a candidate predictor. More marker‐driven prospective studies are warranted.

## Graphical abstract

Immunotherapy has emerged as a promising treatment for GBM patients. The impact of several molecular and clinical variables on survival outcomes of GBM patients treated with immunotherapy has been comprehensively assessed in this systematic review and meta‐analysis. Our findings might help to optimize the immunotherapy efficacy for GBM patients and give insights into grouping patients in clinical trials of immunotherapy for GBM patients.

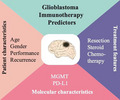

## 1 INTRODUCTION

Glioblastoma multiforme (GBM) is the most common and aggressive central nervous system malignancy with an age‐adjusted incidence of approximately 3–4 per 100,000 population per year.^1^ The median overall survival (OS) of newly diagnosed GBM is about 15–17 months under the standard of care (SOC) including maximal safe surgical resection or a diagnostic biopsy, followed by concurrent chemoradiotherapy and maintenance of temozolomide (TMZ).^2^, ^3^ There is still no SOC for recurrent or progressive GBM. To further prolong the survival and improve the life quality of patients with GBM, a large body of emerging treatment modalities based on the SOC is being investigated, including but not limited to tumor‐treating fields, monoclonal targeted antibodies, small molecule inhibitors, and immunotherapies.^4^, ^5^, ^6^, ^7^ However, despite numerous attempts, no material improvements have been made in the prognosis of patients with GBM.

Immunotherapy, which targets cancer cells through activating the host antitumor immune response, is regarded as a cancer treatment breakthrough.^8^ Furthermore, many clinical trials have been designed to assess the effectiveness of immunotherapies, including immune checkpoint inhibitors (ICI), vaccines, adoptive cellular therapies (ACT), oncolytic viral treatment, and others, for both newly diagnosed and recurrent GBM.^9^, ^10^, ^11^, ^12^ Despite exciting improvements seen for other cancers, many phase III clinical trials of immunotherapy in GBM have failed upon comparing the patients who received this treatment versus those who did not; there remains no FDA‐approved GBM immunotherapy to date.^13^ Of note, a subset of patients in these clinical trials could derive clinical benefits from immunotherapy; thus, uncovering predictive markers that could be used to gauge efficacy before treatment implementation is important to the development of immunotherapy in the treatment of GBM.

The predictive utility of a number of biomarkers for immunotherapy has been identified. Tumor programmed death‐ligand 1 (PD‐L1) expression, tumor‐infiltrating lymphocytes (TILs), and tumor mutation burden (TMB), etc., are used as indicators of clinical efficacy for ICI‐based immunotherapy.^14^ However, studies concerning the predictors of immunotherapy for GBM are limited to date and yield contentious results; thus, there is an urgent need to summarize and uncover robust and effective factors to distinguish which patients would benefit from immunotherapy.^15^, ^16^, ^17^, ^18^

To shed some light on this issue, we conducted a systematic review and meta‐analysis to summarize and test the predictive value of multiple molecular and clinical variables on survival outcomes of GBM patients treated with immunotherapy.

## 2 METHODS

### 2.1 Search strategy

This systematic review and meta‐analysis was performed in accordance with the Preferred Reporting Items for Systematic Reviews and Meta‐Analyses Protocols (PRISMA‐p) 2015 Statement.^19^ The protocol was registered in the Prospective Register of Systematic Reviews (PROSPERO CRD42021284820). We retrieved relevant papers from several electronic databases, including PubMed, EMBASE, Cochrane Library, Web of Science, and ClinicalTrials.gov database, from inception to February 1, 2022, using the following search terms: (“GBM” OR “glioblastoma” OR “glioblastoma multiforme” OR “astrocytoma”) AND (“immunother*” OR “immuno‐oncology” OR “checkpoint inhibitor” OR “checkpoint blockade” OR “PD‐1” OR “PD‐ L1” OR “CTLA‐4” OR “vaccin*” OR “virus” OR “adoptive cell” OR “chimeric antigen receptor” OR “car‐t”) AND (“clinical study” OR “trial”). The reference lists of relevant publications were also checked for potentially eligible studies.

### 2.2 Study selection

Two reviewers independently (Hu and Liu) used eligibility criteria to select and extract data. Disagreements were resolved by discussion with a third reviewer (Chen). The following inclusion criteria were used: (1) studies that enrolled, entirely or partially, patients with histologically confirmed GBM (newly diagnosed or recurrent) treated by immunotherapy; (2) studies that reported on clinical outcomes of OS or progression‐free survival (PFS) with clinical and molecular variables measured before immunotherapy implementation; and (3) randomized controlled trials or non‐randomized trials with at least five patients per group. Excluded studies were those failing to comply with the eligibility criteria and in accordance with the following criteria: (1) studies performed in animals; (2) studies that completely focused on variables detected from patients after immunotherapy; (3) studies that comprised patients with prior receipt of immunotherapy; and (4) clinical trials with insufficient data, observational studies, and reviews, editorials, letters, and case reports. In addition, studies of exploratory or retrospective analysis in relation to predictive factors based on clinical trial data were included, but retrospective observational studies were excluded.

### 2.3 Data extraction

The baseline characteristics of included studies and patients were extracted as follows: authors, publication time, clinical trial design, median follow‐up time, sample size, GBM diagnosis, immunotherapy type, and survival outcomes of the prespecified subgroups. The primary outcome was OS, and the secondary outcome was PFS. The treatment response data were not extracted owing to limited relevant studies. When the necessary data for analysis were not available, we contacted corresponding authors by email to request unpublished data.

### 2.4 Risk‐of‐bias assessments

Quality assessment of randomized controlled trials was performed using RoB 2, a revised tool for measuring the Cochrane risk of bias, which comprises five distinct domains and an overall risk‐of‐bias judgment. It grades studies as “low risk of bias,” “some concerns,” or “high risk of bias.”^20^ For non‐randomized trials, methodological index for non‐randomized studies (MINORS) was utilized, which contains 8 items and 12 items for non‐comparative studies and comparative studies, respectively (scoring 0–2 for each item).^21^

### 2.5 Statistical analysis

Analyses were conducted using R version 4.1.1 (R Core Team). Statistical significance was set at *p* < 0.05. If not available directly, hazard ratio (HR) and 95% confidence interval (CI) for OS or PFS of the individual study were estimated using number of events, *p*‐value, K‐M curve, median survival, or other data from the original study.^22^, ^23^ The data were synthesized by random‐effects model, and variation was estimated using the DerSimonian–Laird method and Jackson method.^24^ Inconsistencies among studies were assessed using an *I* ^2^ quantity and the *Q* test (significant *p* < 0.10).^25^ We also reported stratified pooled outcomes by GBM diagnosis, type of immunotherapy, and study design.

To avoid exaggerated effect sizes and to investigate how each study influenced the overall estimate, we performed a leave‐one‐out analysis. To address publication bias, we used both the visual method of contour‐enhanced funnel plot and the quantitative approach of Eggers' regression to detect publication bias when the included studies were >10.^26^, ^27^ Otherwise, the trim‐and‐fill method was applied to evaluate publication bias.^28^

## 3 RESULTS

### 3.1 Study characteristics and baseline

**FIGURE 1:**
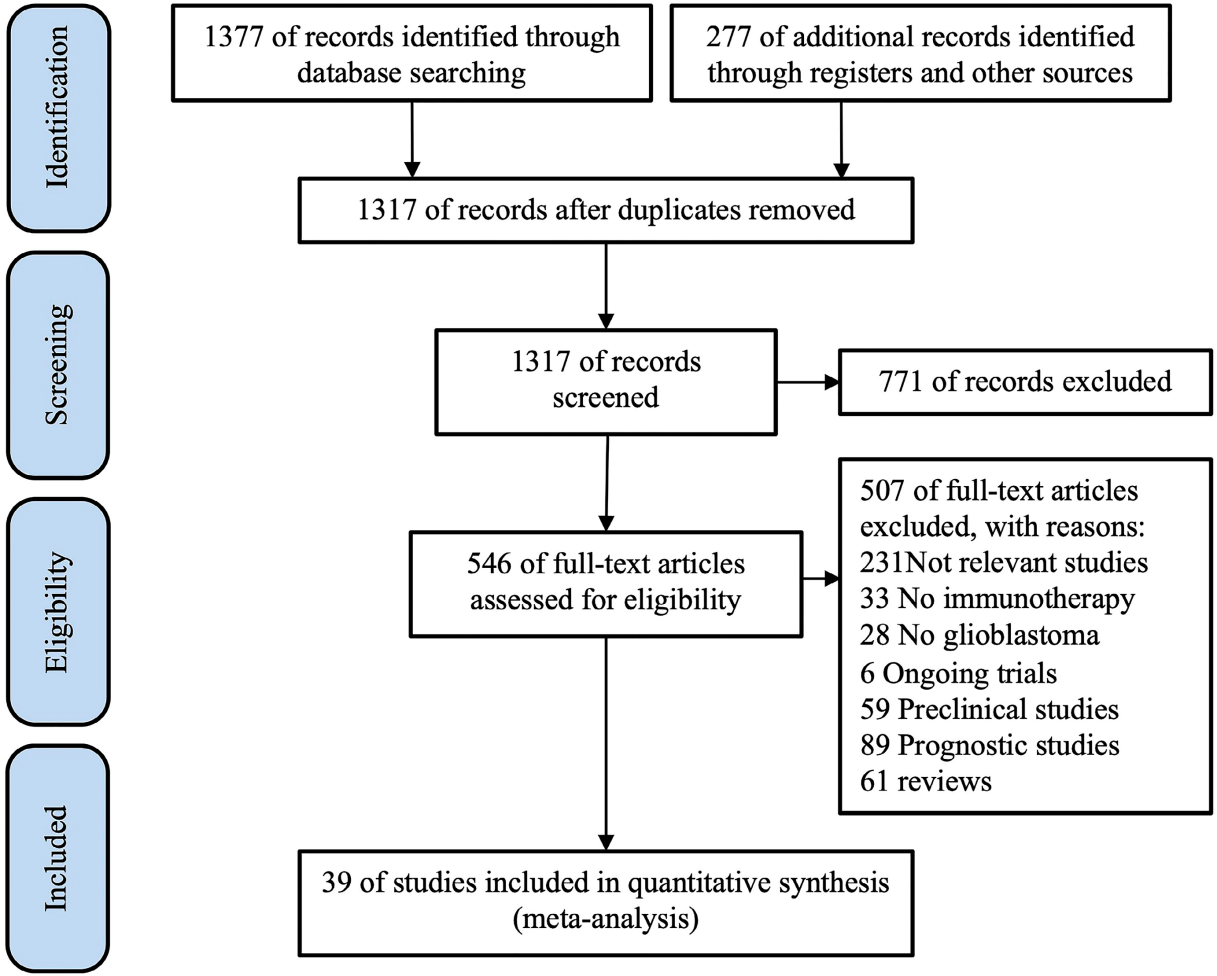
Study selection flow diagram

A total of 1317 papers were identified, and 39 studies were included for quantitative synthesis as shown in the PRISMA flow diagram (Figure 1). The selected 39 studies demonstrated a total of 4488 GBM patients.^10^, ^29^, ^30^, ^31^, ^32^, ^33^, ^34^, ^35^, ^36^, ^37^, ^38^, ^39^, ^40^, ^41^, ^42^, ^43^, ^44^, ^45^, ^46^, ^47^, ^48^, ^49^, ^50^, ^51^, ^52^, ^53^, ^54^, ^55^, ^56^, ^57^, ^58^, ^59^, ^60^, ^61^, ^62^, ^63^, ^64^, ^65^, ^66^ Of the 39 studies, 19 reported on newly diagnosed GBM, 15 reported on recurrent GBM, and 4 included both. The median follow‐up time of included studies ranged from 7.7 to 27.6 months. ICI was implemented in 8 studies; vaccines in 24 studies, including dendritic cell (DC) vaccines and peptide vaccines; ACT in 3 studies; and oncolytic virus treatment in 4 studies. The main baseline characteristics of the included studies are summarized in Table 1. Quality assessment was performed for both included randomized and non‐randomized trials, as shown in Tables S1 and S2.

**TABLE 1:** Characteristics of studies and patients included in the meta‐analysis

Study	Trial	Phase	Randomization	Size (*n*)	median FU (m)	GBM	IMT	Stratification	Outcomes
Reardon et al. (2020)^53^	NCT02017717	III	Yes	369	9.8	Recurrent	ICI–nivolumab	MGMT, resection, KPS, steroid, PD‐L1	OS
Liau et al. (2018)^10^	NCT00045968	III	Yes	331	N/A	Newly	DCs–DCVaxL	MGMT	OS
Nayak et al. (2021)^49^	NCT02337491	II	Yes	80	N/A	Recurrent	ICI–pembrolizumab	MGMT, steroid, KPS, age, gender, resection, PD‐L1, recurrence	OS
Inogés et al. (2017)^42^	NCT01006044	II	No	31	N/A	Newly	DCs–tumor lysate	MGMT, KPS, age, gender, resection	OS
Ardon et al. (2012)^31^	HGG‐2006	I/II	No	77	25	Newly	DCs–tumor lysate	MGMT, resection	OS, PFS
Bloch et al. (2017)^34^	NCT00905060	II	No	46	N/A	Newly	Vaccine–HSPPC‐96	MGMT, KPS	OS, PFS
Ahluwalia et al. (2016)	NCT02455557	II	No	63	21.7	Newly	Vaccine–SurVaxM	MGMT	OS, PFS
Smith et al. (2020)^60^	ACTRN12615000656538	I/II	No	28	N/A	Newly	ACT–CMV‐specific	MGMT, gender	OS
Batich et al. (2017)^32^	NCT00639639	I/II	No	14	N/A	Newly	DCs–CMV pp65	MGMT	OS, PFS
Pellegatta et al. (2018)^50^	DENDR1	II	No	24	17.4	Newly	DCs–tumor lysate	MGMT, steroid, KPS, gender	OS, PFS
Ishikawa et al. (2014)^43^	UMIN000001426	I/IIa	No	24	19.6	Newly	Vaccine–AFTV	MGMT, gender, resection	OS, PFS
Schuster et al. (2015)^59^	ACT III	II	No	81	N/A	Newly	Vaccine–CDX 110	MGMT	OS, PFS
Jan et al. (2018)^45^	N/A	II	Yes	47	N/A	Newly	DCs–tumor lysate	MGMT, gender, resection, PD‐L1, chemotherapy	OS, PFS
Schalper et al. (2019)^58^	NCT02550249	II	No	30	N/A	both	ICI–nivolumab	MGMT, age, gender, resection	OS, PFS
Sampson et al. (2010)^56^	NCT00643097	II	No	18	N/A	Newly	Vaccine–PEPvIII	MGMT, gender, resection, KPS	OS, PFS
Aoki et al. (2021)^30^	JapicCTI‐152,967	II	No	44	N/A	Recurrent	ICI–nivolumab	MGMT, KPS, PD‐L1	OS
Desjardins et al. (2018)^38^	NCT01491893	I	No	61	27.6	Recurrent	OV–PVSRIPO	MGMT, age, recurrence	OS
Izumoto et al. (2008)^44^	N/A	II	No	21	N/A	Recurrent	Vaccine–WT1	steroid, KPS, gender, chemotherapy	OS, PFS
Pellegatta et al. (2013)^51^	N/A	I	No	15	8	Recurrent	DCs–tumor lysate	steroid, gender	OS, PFS
Lukas et al. (2018)^46^	NCT01375842	Ia	No	16	N/A	Recurrent	ICI–atezolizumab	steroid	OS, PFS
Cloughesy et al. (2019)^36^	N/A	N/A	Yes	35	15.6	Recurrent	ICI–pembrolizumab	steroid	OS, PFS
Dillman et al. (2009)^40^	NCT00331526	II	No	33	N/A	Recurrent	ACT–LAK	steroid, chemotherapy	OS
Muragaki et al. (2011)^47^	UMINC000000002	I/IIa	No	24	19	Newly	Vaccine–AFTV	KPS, age, gender, resection	OS, PFS
Geletneky et al. (2017)^41^	NCT01301430	I/IIa	No	18	N/A	Recurrent	OV–ParvOryx	gender, KPS, age	OS, PFS
Takashima et al. (2016)^61^	N/A	II	No	60	N/A	Recurrent	Vaccine–WT1	gender, chemotherapy	OS
Bloch et al. (2013)	NCT00293423	II	No	41	N/A	Recurrent	Vaccine–HSPPC 96	gender	OS
Rudnick et al. (2020)^55^	N/A	I	No	28	N/A	Both	DCs–tumor lysate	gender, resection	OS, PFS
Phuphanich et al. (2013)^52^	N/A	I	No	21	40.1	Both	DCs–ICT 107	gender, resection	OS, PFS
Chiocca et al. (2011)^35^	NCT00751270	Ib	No	13	N/A	Newly	OV‐AdV‐tk	resection	OS, PFS
DiDomenico et al. (2017)^39^	NCT00905060	II	No	46	N/A	Newly	Vaccine–HSPPC‐96	PD‐L1	OS, PFS
Weathers et al. (2020)^63^	NCT02661282	I/II	No	40	12	Both	ACT‐CMV pp65	recurrence	OS
Yao et al. (2018)^66^	N/A	II	Yes	43	14	Both	DCs–GSC antigen	PD‐L1	OS
Reardon et al. (2020b)^54^	NCT01498328	II	Yes	73	N/A	Recurrent	Vaccine–CDX 110	steroid, age, gender, KPS, recurrence	OS
Cloughesy et al. (2020^37^)^a^	NCT02414165	II/III	Yes	403	22.8	Recurrent	OV–Toca	MGMT, resection, steroid, age, gender, KPS, recurrence	OS
Weller et al. (2021)^65^	NCT02667587	III	Yes	716	N/A	Newly	ICI–nivolumab	MGMT, steroid	OS
Weller et al. (2017)^64^	NCT01480479	III	Yes	745	N/A	Newly	Vaccine–CDX 110	MGMT, gender	OS
Ursu et al. (2017)^62^	NCT00190424	II	Yes	81	N/A	Newly	Vaccine–CpG‐ODN	MGMT, KPS	OS
Sampson et al. (2016)^57^	NCT02617589	III	Yes	560	N/A	Newly	ICI–nivolumab	MGMT	OS
Narita et al. (2019)^48^	N/A	III	Yes	88	7.7	Recurrent	Vaccine–PPV	KPS	OS

Abbreviations: ACT, adoptive cell therapy; DCs, dendric cells; FU, follow‐up; GBM, glioblastoma; ICI, immune checkpoint inhibitor; IMT, immunotherapy; KPS, Karnofsky Performance Status; MGMT, O6‐methylguanine‐DNA methyltransferase; N/A, not available/applicable; OS, overall survival; OV, oncolytic virus; PD‐L1, programmed death‐ligand 1; PFS, progression‐free survival.

^a^ This trial contained 12% of anaplastic astrocytoma population.

### 3.2 O‐6‐Methylguanine‐DNA methyltransferase (MGMT) promoter methylation

**FIGURE 2:**
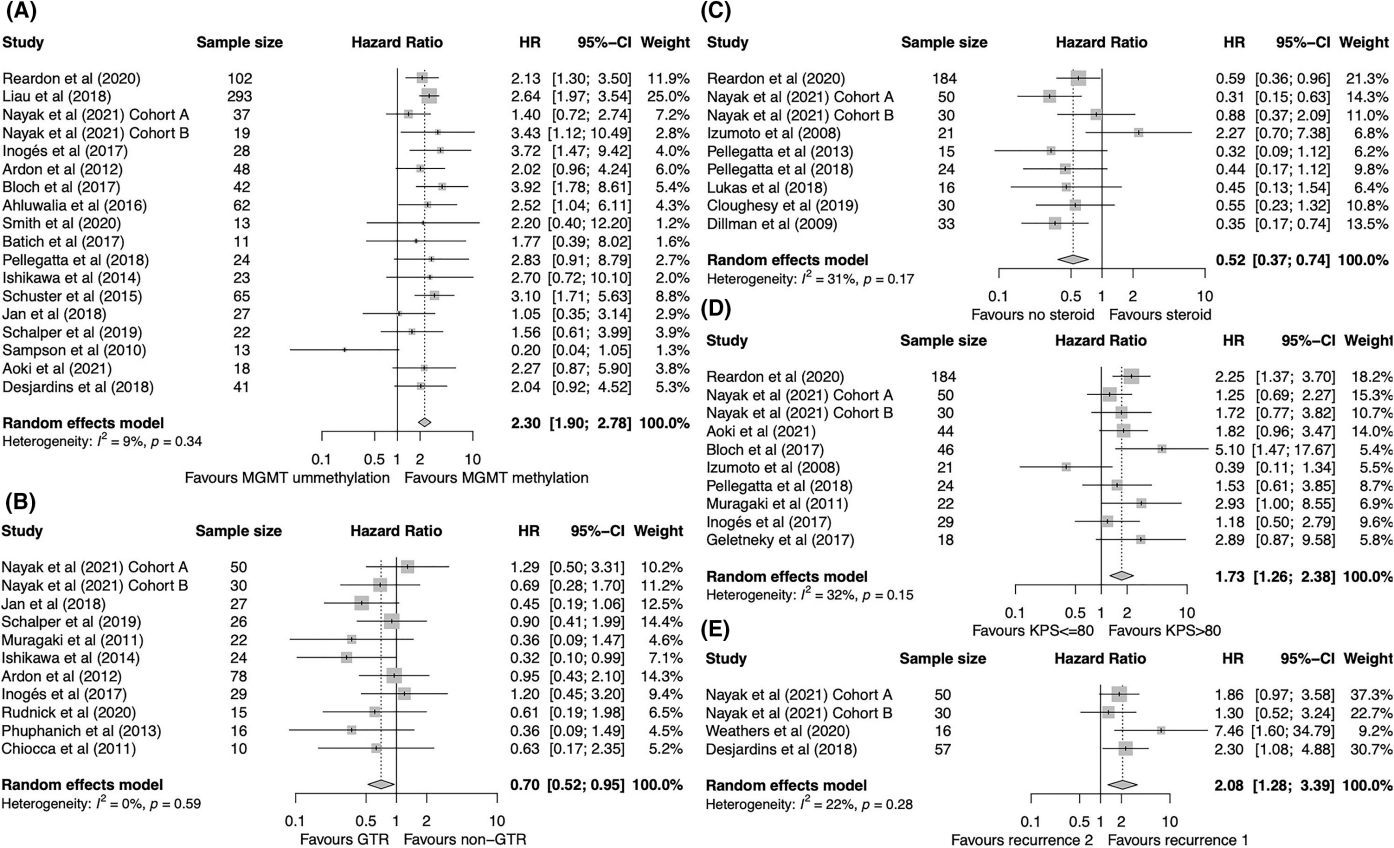
Forest plot of HR for OS of GBM patients treated with immunotherapy according to (A) MGMT promoter methylation status, (B) extent of resection, (C) baseline steroid use, (D) KPS scores, and (E) number of prior recurrences. Gray squares signify point estimates, and square sizes are proportional to study weights. Horizontal lines represent effect size confidence intervals. Diamonds represent pooled effect size; their lengths represent the 95% confidence interval of the pooled estimate. GBM, glioblastoma; HR, hazard ratio; KPS, Karnofsky Performance Status; MGMT, O6‐methylguanine‐DNA methyltransferase; OS, overall survival.

**FIGURE 3:**
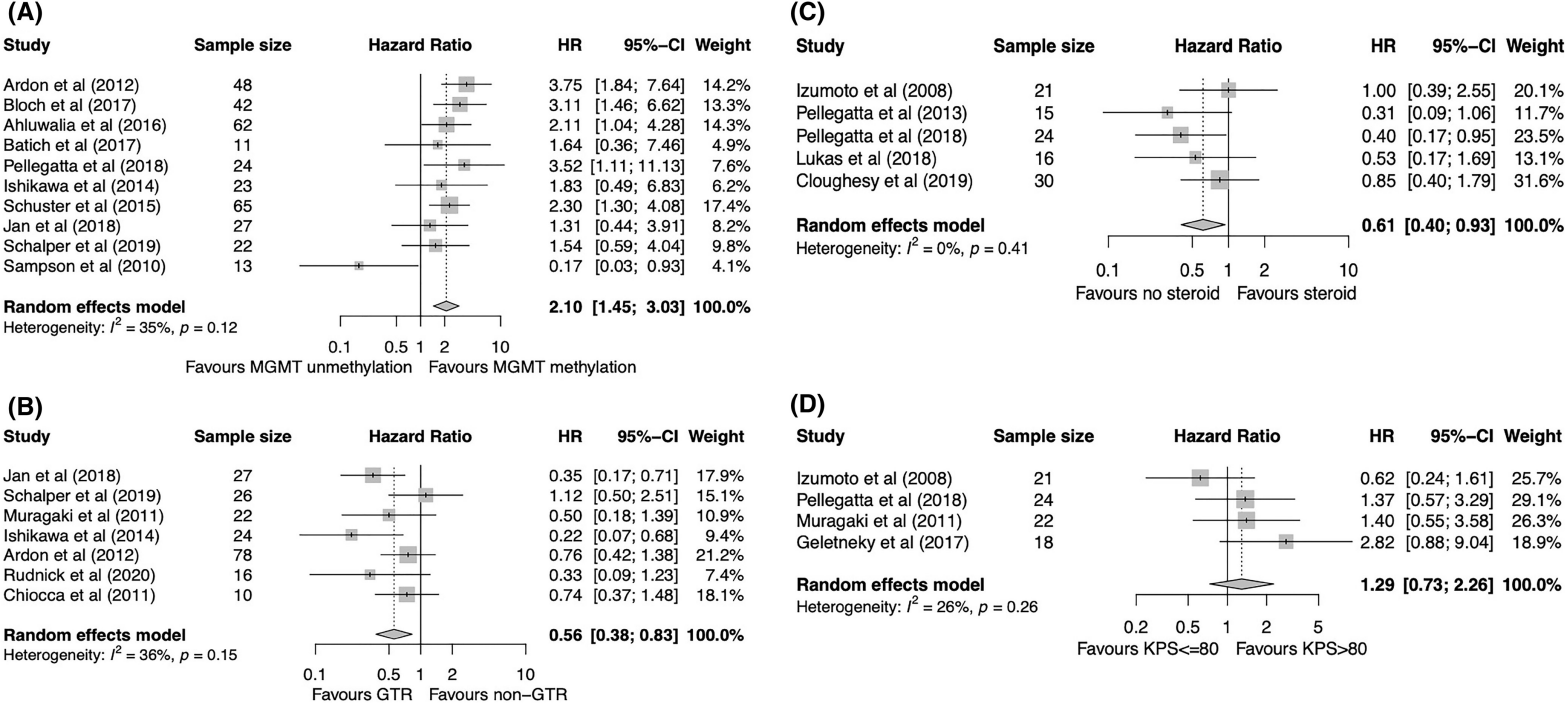
Forest plot of HR for PFS of GBM patients treated with immunotherapy according to (A) MGMT promoter methylation status, (B) extent of resection, (C) baseline steroid use, and (D) KPS scores. Gray squares signify point estimates, and square sizes are proportional to study weights. Horizontal lines represent effect size confidence intervals. Diamonds represent pooled effect size; their lengths represent the 95% confidence interval of the pooled estimate. GBM, glioblastoma; HR, hazard ratio; KPS, Karnofsky Performance Status; MGMT, O6‐methylguanine‐DNA methyltransferase; PFS, progression‐free survival.

Pooling results from 18 cohorts demonstrated a statistically significant mortality disadvantage of carrying MGMT promoter unmethylation versus MGMT promoter methylation (HR = 2.30, 95% CI: 1.90–2.78, *p* < 0.0001) among GBM patients receiving immunotherapy (Figure 2A). Stratified analysis by different baseline characteristics showed a consistent correlation of MGMT promoter unmethylation with a worse OS in all subgroups (Table 2). Furthermore, meta‐analysis of 10 cohorts indicated a significant association of worse PFS with MGMT promoter unmethylation versus methylation (HR = 2.10, 95% CI: 1.45–3.03, *p* < 0.0001) (Figure 3A). In addition, eight cohorts explored the efficacy of immunotherapy over non‐immunotherapy among patients with different MGMT promoter methylation statuses. When compared to the non‐immunotherapy control group, no significant association was seen between the use of immunotherapy and OS among patients with MGMT methylation (HR = 0.79, 95% CI: 0.53–1.19) and those without MGMT methylation (HR = 1.14, 95% CI: 0.97–1.33) (Figure S1A). However, a trend was seen in OS difference in that immunotherapy seemed to predict a better OS in the MGMT methylation subgroup, although it did not reach significance (*p* = 0.10) (Figure S1A).

**TABLE 2:** The results of subgroup analysis for overall survival of patients treated with immunotherapy

Population	Subgroup	No. cohorts	Meta‐analysis		Test for heterogeneity	
			HR	95%CI	*I* ^2^	*p*‐Value
Unmet‐MGMT vs. met‐MGMT	Total	18	**2.30**	**1.90–2.78**	9%	0.34
	nGBM	12	**2.44**	**1.84–3.23**	24%	0.21
	rGBM	6	**1.96**	**1.45–2.65**	0%	0.8
	ICI	6	**2.17**	**1.63–2.89**	0%	0.53
	Vaccine	10	**2.30**	**1.62–3.25**	36%	0.12
	Randomized	5	**2.15**	**1.58–2.92**	27%	0.24
	Non‐randomized	13	**2.39**	**1.82–3.15**	9%	0.36
GTR vs. no GTR	Total	11	**0.70**	**0.52–0.95**	0%	0.6
	nGBM	7	**0.61**	**0.41–0.90**	0%	0.44
	rGBM	4	0.86	0.54–1.37	0%	0.73
	ICI	3	0.92	0.56–1.52	0%	0.64
	Vaccine	7	**0.61**	**0.41–0.90**	0%	0.45
	Randomized	3	0.72	0.40–1.30	24%	0.27
	Non‐randomized	8	0.70	0.48–1.01	0%	0.57
Baseline no steroid vs. Steroid	Total	9	**0.52**	**0.37–0.74**	31%	0.17
	nGBM	1	0.44	0.17–1.12	N/A	N/A
	rGBM	8	**0.54**	**0.37–0.79**	39%	0.12
	ICI	5	**0.53**	**0.38–0.73**	0%	0.44
	Vaccine	3	0.68	0.22–2.11	68%	0.04
	Randomized	4	**0.54**	**0.36–0.79**	19%	0.29
	Non‐randomized	5	0.53	0.27–1.01	49%	0.1
KPS ≤80 vs. >80	Total	10	**1.73**	**1.26–2.38**	32%	0.15
	nGBM	4	**2.05**	**1.11–3.78**	32%	0.22
	rGBM	6	**1.61**	**1.08–2.39**	41%	0.13
	ICI	4	**1.77**	**1.31–2.40**	0%	0.53
	Vaccine	5	1.59	0.76–3.35	60%	0.04
	Randomized	3	**1.75**	**1.21–2.52**	9%	0.33
	Non‐randomized	7	**1.75**	**1.06–2.89**	45%	0.09
Prior recurrence twice vs. once	Total	4	**2.08**	**1.28–3.99**	0.22	0.28
	nGBM	0	N/A	N/A	N/A	N/A
	rGBM	4	**2.08**	**1.28–3.99**	22%	0.28
	ICI	2	1.65	0.97–2.81	0%	0.53
	Vaccine	0	N/A	N/A	N/A	N/A
	Randomized	2	1.65	0.97–2.81	0%	0.53
	Non‐randomized	2	**3.39**	**1.15–10.03**	45%	0.18
Male vs. Female	Total	16	0.92	0.73–1.17	0%	0.62
	nGBM	8	0.94	0.65–1.36	0%	0.68
	rGBM	8	0.90	0.65–1.26	11%	0.34
	ICI	3	1.03	0.67–1.59	0%	0.43
	Vaccine	11	0.88	0.65–1.20	6%	0.39
	Randomized	3	0.98	0.63–0.51	2%	0.36
	Non‐randomized	13	0.90	0.67–1.19	0%	0.57
Age ≥65 vs. <65	Total	7	1.25	0.88–1.78	0%	0.82
	nGBM	2	**1.92**	**1.01–3.66**	0%	0.54
	rGBM	5	1.04	0.68–1.59	0%	1
	ICI	3	1.07	0.64–1.78	0%	0.99
	Vaccine	2	**1.92**	**1.01–3.66**	0%	0.54
	Randomized	2	1.07	0.58–1.96	0%	0.89
	Non‐randomized	5	1.35	0.88–2.09	0%	0.65
Tumor PD‐L1+ vs. PD‐L1‐	Total	5	1.1	0.66–1.84	63%	0.03
	nGBM	2	0.74	0.25–2.18	67%	0.08
	rGBM	3	1.36	0.62–2.98	70%	0.03
	ICI	3	1.36	0.62–2.98	70%	0.03
	Vaccine	2	0.74	0.25–2.18	67%	0.08
	Randomized	3	0.81	0.51–1.29	2%	0.36
	Non‐randomized	2	1.75	0.66–4.61	82%	0.02
Chemotherapy vs. No chemotherapy	Total	4	1.04	0.46–2.33	65%	0.04
	nGBM	1	1.06	0.44–2.57	N/A	N/A
	rGBM	3	1.04	0.32–3.39	76%	0.01
	ICI	0	N/A	N/A	N/A	N/A
	Vaccine	3	1.51	0.86–2.65	0%	0.41
	Randomized	1	1.06	0.44–2.57	N/A	N/A
	Non‐randomized	3	1.04	0.32–3.39	76%	0.01

Abbreviations: CI, confidence interval; GTR, gross total resection; HR, hazard ratio; ICI, immune checkpoint inhibitor; KPS, Karnofsky Performance Status; met, promoter methylation; MGMT, O6‐methylguanine‐DNA methyltransferase promoter methylation; N/A, not available/applicable; nGBM, newly diagnosed glioblastoma; No., number; PD‐L1, Programmed death‐ligand 1; rGBM, recurrent glioblastoma; unmet, promoter unmethylation.

### 3.3 Gross total resection

A total of 11 cohorts reported on the association between OS and the extent of resection in GBM patients receiving immunotherapy. Pooled results showed that gross total resection (GTR) was associated with a statistically significant better OS in comparison with the non‐GTR group (HR = 0.70, 95% CI: 0.52–0.95, *p* = 0.02) (Figure 2B). However, inconsistent results from further subgroup analysis revealed that only in the subsets of patients with newly diagnosed GBM and vaccine‐based immunotherapy, was GTR significantly associated with a better OS (Table 2). By pooling the results of seven cohorts reporting on PFS, GTR was also associated with a significantly prolonged PFS compared with that of the non‐GTR group (HR = 0.56, 95% CI: 0.38–0.83, *p* = 0.004) (Figure 3B). In addition, three randomized trials were included for meta‐analysis of the immunotherapy experiment group versus non‐immunotherapy control group. Immunotherapy did not significantly improve OS in GTR patients (HR = 0.62, 95% CI: 0.25–1.54) nor in non‐GTR patients (HR = 0.60, 95% CI: 0.28–1.30) in comparison with non‐immunotherapy, but a significant heterogeneity was observed (*Q* test, *p* < 0.01) (Figure S1B).

### 3.4 Baseline steroid use

Meta‐analysis of nine cohorts demonstrated that the non‐steroid group correlated with a significantly favorable OS in comparison with that of the steroid group in GBM patients treated with immunotherapy (HR = 0.52, 95% CI: 0.37–0.74, *p* = 0.0002) (Figure 2C). Stratified analysis showed that no significant association was observed in subgroups of patients with newly diagnosed GBM, in those receiving a vaccine, and in non‐randomized studies (Table 2). In terms of PFS, a significant association between the absence of baseline steroid use and improved PFS was observed (HR = 0.61, 95% CI: 0.40–0.93, *p* = 0.02) (Figure 3C). Additionally, four randomized studies investigated the OS of patients treated with immunotherapy according to baseline steroid use. No significant difference was found in OS of the immunotherapy group over the control group among patients without (HR = 0.94, 95% CI: 0.73–1.22) or with (HR = 1.01, 95% CI: 0.66–1.54) baseline steroid use (Figure S1C).

### 3.5 Karnofsky Performance Status

A total of 10 cohorts reported on the association between OS and Karnofsky Performance Status (KPS) score in GBM patients treated with immunotherapy, and the combined results showed a significant disadvantageous OS in patients with KPS ≤80 versus those with KPS > 80 (HR = 1.73, 95% CI: 1.26–2.38, *p* = 0.0007) (Figure 2D). The subgroup analysis demonstrated that the significant association was retained in all subsets except for the subgroup receiving the vaccine (Table 2). After combining the results of five cohorts reporting on PFS, no significant difference in PFS was observed between patients with a KPS ≤80 and >80 (HR = 1.29, 95% CI: 0.73–2.26) (Figure 3D). In the further meta‐analysis of immunotherapy versus control, integrating results from four studies, a favorable OS was not achieved by immunotherapy compared with the control treatment, irrespective of KPS scores according to ≤80 and >80 (Figure S1D).

### 3.6 Number of prior relapses

**FIGURE 4:**
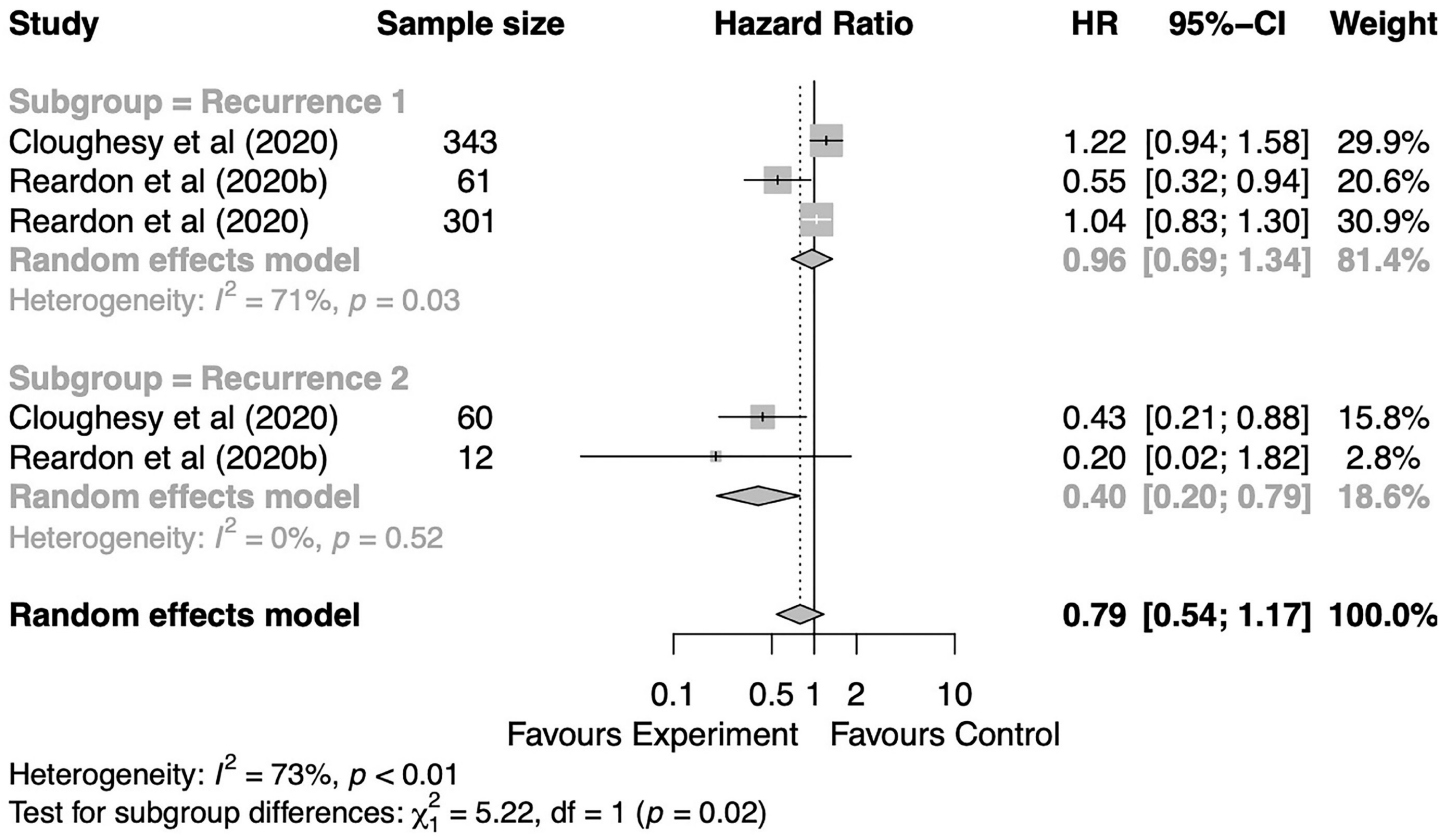
Forest plot of HR for OS of GBM patients with GBM comparing immunotherapy and non‐immunotherapy control groups based on number of prior recurrences. Gray squares signify point estimates, and square sizes are proportional to study weights. Horizontal lines represent effect size confidence intervals. Diamonds represent pooled effect size; their lengths represent the 95% confidence interval of the pooled estimate. GBM, glioblastoma; HR, hazard ratio; OS, overall survival.

Pooling results of four cohorts reporting on recurrence frequency in GBM patients treated with immunotherapy showed a significantly worse OS of patients who had a previous recurrence twice versus once (HR = 2.08, 95% CI: 1.28–3.39, *p* = 0.003) (Figure 2E); no significant heterogeneity was observed (*I* ^2^ = 22%, *Q* test, *p* = 0.28). Remarkably, immunotherapy significantly improved OS in patients with two prior recurrences (HR = 0.40, 95% CI: 0.20–0.79, *p* = 0.008), but not in patients with one prior recurrence (HR = 0.96, 95% CI: 0.69–1.34) compared with the control treatment, although only two studies were available for this analysis (Figure 4).

### 3.7 Tumor PD‐L1 expression

A total of five cohorts reported on the tumor PD‐L1 expression in GBM patients treated with immunotherapy, including 5 for OS and 2 for PFS. The pooled results showed a nonsignificant difference in OS (HR = 1.10, 95% CI: 0.66–1.84; Figure S2A) and PFS (HR = 0.86, 95% CI: 0.39–1.90; Figure S3A) when comparing PD‐L1‐positive and PD‐L1‐negative groups. A nonsignificant association was observed in all subgroups (Table 2). Three randomized clinical trials explored the efficacy of immunotherapy versus the control in patients with different tumor PD‐L1 expression statuses. The pooled results did not reach a significant difference in OS, both among tumor PD‐L1‐positive patients (HR = 0.40, 95% CI: 0.08–1.94) and tumor PD‐L1‐negative patients (HR = 0.47, 95% CI: 0.17–1.31); a significant heterogeneity was observed (*Q* test, *p* < 0.01) (Figure S4A).

### 3.8 Age, gender, and chemotherapy history

By pooling 7 cohorts reporting on OS and 3 cohorts reporting on PFS for age, we found no significant prognostic value of age (≥65 years of age vs. <65 years of age) for OS (Figure S2B) and PFS (Figure S3B) of patients treated with immunotherapy. Of note, stratified analysis revealed that the subset of patients with newly diagnosed GBM receiving a vaccine and aged ≥65 had a significant inferior OS in comparison with those aged <65 (HR = 1.92, 95% CI: 1.01–3.66; Table 2). Additionally, immunotherapy did not significantly improve OS both among patients aged ≥65 and patients aged <65 (Figure S4B).

No significant differences in OS (Figure S2C) or PFS (Figure S3C) between GBM patients treated with immunotherapy stratified by gender were observed, and there was no significant difference in OS of immunotherapy over the control (Figure S4C). The history of chemotherapy also did not impact the OS of patients treated with immunotherapy (Figure S2D).

### 3.9 Publication bias and sensitivity analysis

According to the contour‐enhanced funnel plots (Figures S5 and S6), no visually obvious asymmetry was observed suggesting the possibility of publication bias was low. Meanwhile, Egger's regression test was performed when the meta‐analysis contained >10 studies, and no significant publication bias was found.

The leave‐one‐out analysis demonstrated that the significance of the pooled effects did not substantially depend on any single study (Figure S7). However, the combined effects were not robust enough in both the pooled HR for PFS of baseline steroid use and the pooled HR for OS of GTR.

## 4 DISCUSSION

This study was aimed at identifying the factors that indicate the prognosis of immunotherapy for GBM and exploring the potential predictors with the ability to identify the patients most likely benefit from this therapy. To our knowledge, this is the first systematic review to address this issue. Immunotherapy has been regarded as a breakthrough in the treatment of cancers, and utilizes ICI, vaccines, ACT, oncolytic viral treatment, and other modalities, for the treatment of both newly diagnosed and recurrent GBM.^9^, ^10^, ^11^, ^12^ New types of immunotherapies have been emerging. For instance, antibodies against CD47 or CD24 have been proposed as new targets for glioblastoma therapy.^67^ Preclinical studies have revealed the potential of dual targeting of IL‐6 and CD40 or a combination of IL‐12 and CAR‐T immunotherapy.^68^, ^69^ These could be future clinical therapies for glioblastoma.

Pooling and analysis of the available literature suggest that GBM patients with MGMT promoter methylation, GTR, no baseline steroid use, a KPS > 80, and undergoing one prior recurrence had a significantly relative favorable OS following immunotherapy. In addition, patients receiving immunotherapy with MGMT promoter methylation, GTR, and no baseline steroid use also had a relatively prolonged PFS. As compared to the non‐immunotherapy control group, immunotherapy significantly improved OS among patients with two prior recurrences, but not other subgroups. Taken together, this study indicates that GBM patients with GTR, MGMT promoter methylation, and no baseline steroid use could gain more survival benefits following immunotherapy; patients undergoing two prior recurrences may obtain more benefits from immunotherapy over non‐immunotherapy. Additionally, several potential predictive factors were reviewed qualitatively (Table 3).

**TABLE 3:** Qualitative analysis of other predictors for glioblastoma immunotherapy from relevant clinical studies

Study	Design	GBM	IMT	Predictor	Outcomes
Patient clinical characteristics					
Ishikawa et al. (2014)^43^	Phase I/IIa	nGBM	Vaccine	RPA (III/IV vs. V)	OS
Ardon et al. (2012)^31^	Phase I/II	nGBM	DCs	RPA	OS, PFS
Narita et al. (2019)^48^	Phase III	rGBM	Vaccine	age (≥70 vs. <70 years old), weight (≥70 vs. <70 kg), PS (0–2 vs. 3)	OS
Tumor mutational burden and signatures					
Bouffet et al. (2016)^91^	Case report	rGBM	ICI	germline bMMRD with hypermutation	response
Johanns et al. (2016)^92^	Case report	rGBM	ICI	germline POLE alteration with hypermutation	response
AlHarbi et al. (2018)^93^	Case report	rGBM	ICI	constitutional bMMRD	response
Gromeier et al. (2021)^94^	Retrospective	rGBM	ICI, OV	tumor mutational burden	OS
Zhao et al. (2019)^95^	Retrospective	rGBM	ICI	MAPK pathway alterations (PTPN11, BRAF); PTEN mutation	response
Yao et al. (2018)	Phase II	both	DCs	TERT mutation	OS, PFS
Tumor molecular characteristics					
Arrieta et al. (2021)^108^	Retrospective	rGBM	ICI	ERK1/2 phosphorylation	OS
Ishikawa et al. (2007)^101^	pilot clinical trials	both	vaccine	p53 and MHC‐1 expression	response
Liau et al. (2005)^102^	Phase I	both	DCs	TGF‐2 expression	OS
Yao et al. (2018)^66^	Phase II	both	DCs	B7‐H4 protein expression	OS
Duerinck et al. (2021)^100^	Phase I	rGBM	ICI	B7‐H3 mRNA expression	OS
Chiba et al. (2010)^103^	Retrospective	rGBM	Vaccine	intermediate WT1 expression	OS, PFS
Phuphanich et al. (2013)^52^	Phase I	nGBM	Vaccine	mRNA expression of vaccine‐targeted tumor antigen	OS, PFS
Prins et al. (2011)^107^	Phase I	both	DCs	mesenchymal gene expression profile	OS
Tumor‐infiltrating lymphocytes					
Jan et al. (2018)^45^	Phase II	nGBM	DCs	low PD‐1+/CD8+ ratio on TILs and PBMCs; low PD‐l + TILs	OS, PFS
Zhang et al. (2020)^115^	Retrospective	nGBM	Vaccine	low TCR repertoire diversity; TCR clones of TILs;	OS
Hsu et al. (2016)^114^	Retrospective	both	DCs	higher estimated TIL content	OS, PFS
Patient peripheral blood					
Lukas et al. (2018)^46^	Phase I	rGBM	ICI	high baseline peripheral CD4+ T cells and B cells	OS, PFS
Erhart et al. (2018)^116^	Phase II	nGBM	DCs	GranzB production; CD8+ T cells counts	OS
Bloch et al. (2017)^34^	Phase II	nGBM	Vaccine	PD‐L1 expression on myeloid cells	OS, PFS
Narita et al. (2019)^48^	Phase III	rGBM	Vaccine	CD11b + CD14 + HLA‐DR^low^ monocytes; CCL2 in plasma; CD3 + CD4 + CD45RA − T cells	OS
Takashima et al. (2016)^61^	Phase II	rGBM	Vaccine	low SDC‐4 mRNA expression levels of PBMCs	OS

Abbreviations: bMMRD, biallelic mismatch repair deficiency; DCs, dendric cells; GBM, glioblastoma; ICI, immune checkpoint inhibitor; IMT, immunotherapy; nGBM, newly diagnosed GBM; OS, overall survival; OV, oncolytic virus; PBMCs, peripheral blood mononuclear cells; PD‐1, programmed cell death protein 1; PD‐L1, programmed death‐ligand 1; PFS, progression‐free survival; rGBM, recurrent GBM; RPA, recursive partitioning analysis; TCR, T‐cell receptor; TILs, tumor‐infiltrating lymphocytes; WT1, Wilms' tumor 1.

### 4.1 Impact of patient clinical characteristics

Intriguingly, we found that having two prior recurrences was an inferior prognostic indicator for patients treated with immunotherapy; however, patients undergoing two previous relapses predicted a significantly superior OS for immunotherapy, as compared to the control treatment. Schulte et al.^70^ reported genetic events following neuroblastoma recurrence that resulted in significantly increased single‐nucleotide variants, indicating tumor transformation and clone selection at recurrence after treatment. Wang et al.^71^ reported that a fraction of GBM patients relapsed after chemotherapy with hypermutation, which was regarded as a predictor for immunotherapy. Accordingly, we conjectured that multiple recurrences may increase the TMB and thus confer more sensitivity on tumors to immunotherapy. However, the interpretation of the predictive value of recurrences should be made with caution given the limited studies included in this analysis.

One previous study retrospectively analyzed the impact of age on the efficacy of immunotherapy for recurrent GBM. They found a positive correlation between patients aged ≥65 years and reduced OS, but not with those aged <65 years; advanced age could suppress immune activity in the brain.^72^ In addition, a phase III clinical trial of a peptide vaccine for recurrent GBM showed that, among patients aged ≥70 years, the immunotherapy group was associated with a disadvantageous OS compared with that of the placebo group.^48^ Our study indicated that patients with newly diagnosed GBM aged ≥65 years were significantly associated with decreased OS compared with those aged <65 years, but this effect was not seen in other subgroups; no significant difference in efficacy of immunotherapy versus placebo was observed among patients aged <65 or ≥ 65 years. All of these results suggested that old age was an unfavorable prognostic factor for patients treated with immunotherapy. A recent meta‐analysis reported more treatment benefits of immunotherapy, particularly CTLA‐4 inhibitors, in men than in women with advanced cancers, excluding melanoma.^73^ In the present study for GBM, however, no difference in efficacy of immunotherapy was associated with gender. Analysis of the potential association of other demographic variables with the immunotherapeutic efficacy merits further studies, such as weight ≥70 kg presumably acting as an unfavorable indicator of immunotherapy for GBM.^48^

The relationship of performance status (PS) measured by several scales, such as KPS or Eastern Cooperative Oncology Group PS, has also been investigated. Several meta‐analyses explored the association of PS with immunotherapy efficacy in multiple advanced malignancies but not GBM; they found that immunotherapy efficacy did not differ among patients according to PS scores.^74^, ^75^ Narita et al.^48^ reported that a performance score of PS 3 was an unfavorable factor for immunotherapy efficacy for recurrent GBM. Our meta‐analysis results indicated that patients with a KPS > 80 seemed to have a better OS after immunotherapy; yet, it failed to predict a better OS for the immunotherapy group than for the control group.

### 4.2 Impact of treatment features

A retrospective single‐center prognostic analysis performed by Ishikawa et al. showed that among patients with newly diagnosed GBM receiving immunotherapy, GTR was a good prognostic factor associated with better OS and PFS.^76^ Similarly, our results demonstrated the consistent prognostic value of GTR for the survival of GBM patients receiving immunotherapy, yet GTR did not predict a better OS for the immunotherapy group versus the control group. Nevertheless, according to these results, GTR might be recommended before immunotherapy.

The use of chemotherapy for GBM patients may result in lymphopenia, including decreased total effector T cells and an increased proportion of T‐reg cells, but the administration of chemotherapy may not exert a negative effect on the immunotherapeutic efficacy.^77^, ^78^, ^79^ In addition, researchers found that nature killer cells could escape apoptosis from chemotherapy and contribute to antitumor immunity.^50^, ^80^

In terms of steroid use, corticosteroids are among the most effective agents for central nervous system tumors in relieving brain edema and palliating neurological deficits.^81^ However, due to a well‐known immunosuppressive effect, corticosteroids are believed to compromise the efficacy of immuno‐oncology.^82^ Arbour et al. reported that baseline corticosteroid use correlated with worse OS, PFS, and the overall response rate in patients with non‐small‐cell lung cancer treated with PD‐(L)1 inhibitors.^83^ Likewise, in the present meta‐analysis, baseline steroid administration was associated with decreased OS and PFS in patients with GBM treated with immunotherapy, suggesting a prudent choice of steroid use preceding immunotherapy for GBM.

### 4.3 Impact of tumorous characteristics

The TMB has been proposed as a predictive biomarker of immunotherapy for a subset of cancer types.^84^, ^85^, ^86^ Higher TMB was believed to produce more mutant tumor neoantigen and further elicit a neoantigen‐specific immune response, which largely accounts for its utility in immunotherapy prediction.^87^ However, the role of TMB in GBM immunotherapy has yet to be well established. GBM, unlike other cancers harboring a mutation signature induced by carcinogens, exhibits a relatively low presence of hypermutation.^88^, ^89^ Two hypermutation pathways were described by Touat et al. in association with GBM: one is a de novo pathway that correlates with constitutive gene defects, and the other is a post‐treatment pathway that is associated with recurrences after chemotherapy.^90^ Of note, germline mutations in DNA polymerase and replication repair gene correlated with ultra‐hypermutation and good responses to immunotherapy in several case reports.^91^, ^92^, ^93^ However, Gromeier et al. recently reported that patients with recurrent GBM with low mutation burden had a predicted longer survival after oncolytic viral treatment or ICI treatment, suggesting the predictive value of a low tumor mutational burden for recurrent GBM.^94^ In addition, some tumor mutational signatures were reported to correlate with immunotherapy efficacy in GBM, such as PTEN mutation, MAPK pathway‐associated gene alteration, and TERT mutation.^66^, ^95^

Tumor PD‐L1 (B7‐H1) expression, measured by immunohistochemistry, has been shown to be a predictive biomarker of the response to anti‐PD‐L1 therapy, especially in melanoma and non‐small‐cell lung cancer.^96^ Although the presence of PD‐L1 expression is not rare in GBM, ranging from 61% to 88%, its role in predicting GBM survival outcomes remains elusive.^97^, ^98^ Meanwhile, studies investigating the PD‐L1 predictive value in the response of GBM to immunotherapy are limited.^99^ The present analysis revealed that tumor PD‐L1 was not associated with prognostic outcomes of patients with GBM treated with immunotherapy and did not predict a better outcome following immunotherapy over control therapy, suggesting the limited predictive value of tumor PD‐L1 expression. Additionally, the predictive roles of other molecules, such as B7‐H3, B7‐H4, TGF‐2, p53, MHC‐1, WT1, and tumor antigens, have also been investigated in several studies.^52^, ^66^, ^100^, ^101^, ^102^, ^103^

MGMT promoter methylation, which is the epigenetic silencing of DNA repair‐associated gene MGMT, has proven to be an independent favorable prognostic factor and predictive of responses to TMZ for GBM.^104^, ^105^, ^106^ Noteworthy is the association between MGMT promoter methylation and the appearance of hypermutation at recurrence after chemotherapy in GBM.^75^, ^90^ Here, we found that MGMT promoter methylation correlated with a favorable prognosis of patients treated with immunotherapy; yet, immunotherapy did not significantly ameliorate OS among patients harboring methylated MGMT promoter or unmethylated promoter, compared with the control treatment. This suggests a limited value of MGMT promoter methylation in guiding patient treatment choice for immunotherapy.

In addition, Erk1/2 phosphorylation and mesenchymal gene expression profiles of GBM are potentially promising markers for immunotherapeutic prediction.^107^, ^108^ Besides, few molecules have been identified to be related to GBM prognosis and chemoresistance, including caveolae‐associated protein 1, B2M, CXCL1, TRIB2, MAP3K1, and Paxillin. These molecules are potential candidate biomarkers to predict glioblastoma immunotherapy.^109^, ^110^, ^111^, ^112^, ^113^

### 4.4 Impact of TILs and peripheral blood biomarkers

Jan et al. reported that a low PD‐1+/CD8+ ratio of TILs or peripheral blood lymphocytes correlated with a better survival outcome among patients with GBM treated with DC vaccines.^45^ The T‐cell receptor repertoire and estimated TIL content also correlate with survival outcomes following immunotherapy for GBM.^114^, ^115^ CD4+ T cell, CD8+ T cell, B cell, and other subtypes of lymphocytes in peripheral blood have been shown to be associated with survival outcome following immunotherapy.^46^, ^48^, ^116^ Additionally, peripheral PD‐L1 expression and SDC‐4 expression correlated with immunotherapeutic efficacy in several studies.^34^, ^61^

In addition, noninvasive MRI technologies have also been recently reported to serve as useful approaches to predict immunotherapy responses in GBM patients. Hagiwara et al. and Cuccarini et al. have reported that MRI relative apparent diffusion coefficient (rADC) may be an imaging biomarker for predicting survival benefits in GBM patients administered with immunotherapies.^117^, ^118^ Additionally, tumor microenvironment alterations in oxygen metabolism, neovascularization, and energy metabolism are critically implicated in therapy failure and recurrence of glioblastoma, which can be detected using the MRI approach and could thus be associated with glioblastoma recurrence and therapy resistance.^119^, ^120^

Our meta‐analysis has some limitations. First, the papers included 13 randomized clinical trials and 26 non‐randomized clinical trials, which might give rise to potential confounding factors, although we did conduct a subgroup analysis stratified by study type. Second, our analysis depended on published study‐level data rather than individual‐patient data, which prevented us from further study of different ages and KPS cutoffs. Third, our study could not fully address the question of which patients most likely benefit from immunotherapy, due to limited randomized control trials involved in this field to date. Therefore, our interpretations of outcomes should be considered with care.

## 5 CONCLUSION

Our analysis found that GBM patients with MGMT promoter methylation, GTR, and no baseline steroid use had favorable prognostic survival outcomes following immunotherapy. No association was observed for age, gender, or tumor PD‐L1 expression with survival outcomes of patients treated with immunotherapy. Furthermore, immunotherapy significantly ameliorated OS compared with non‐immunotherapy among patients undergoing two prior recurrences but not among any other subgroups, suggesting patients with more than one relapse were more likely to derive benefits from immunotherapy. The results from this study may help inform prognostic outcomes of GBM patients treated with immunotherapy and give insights into optimizing immunotherapy efficacy for GBM, as well as provide inferences in grouping GBM patients in clinical studies of immunotherapy. Nevertheless, more marker‐driven prospective clinical trials are warranted to identify and confirm more effective and robust predictive factors of immunotherapy in the treatment of GBM.

## CONFLICT OF INTEREST

The authors declare no conflicts of interest.

## Supporting information


Figure S1

Figure S2

Figure S3

Figure S4

Figure S5

Figure S6

Figure S7

Table S1

Table S2

## Data Availability

All data associated with this study are present in the paper or the Supplementary Materials.
