# Supplementary figures and images for: Impact of molecular and clinical variables on survival outcome with immunotherapy for glioblastoma patients: A systematic review and meta‐analysis
Source: CNS Neurosci Ther. 2022 Jul 13;28(10):1476–91. doi: 10.1111/cns.13915 (PMC9437230; doi:10.1111/cns.13915)

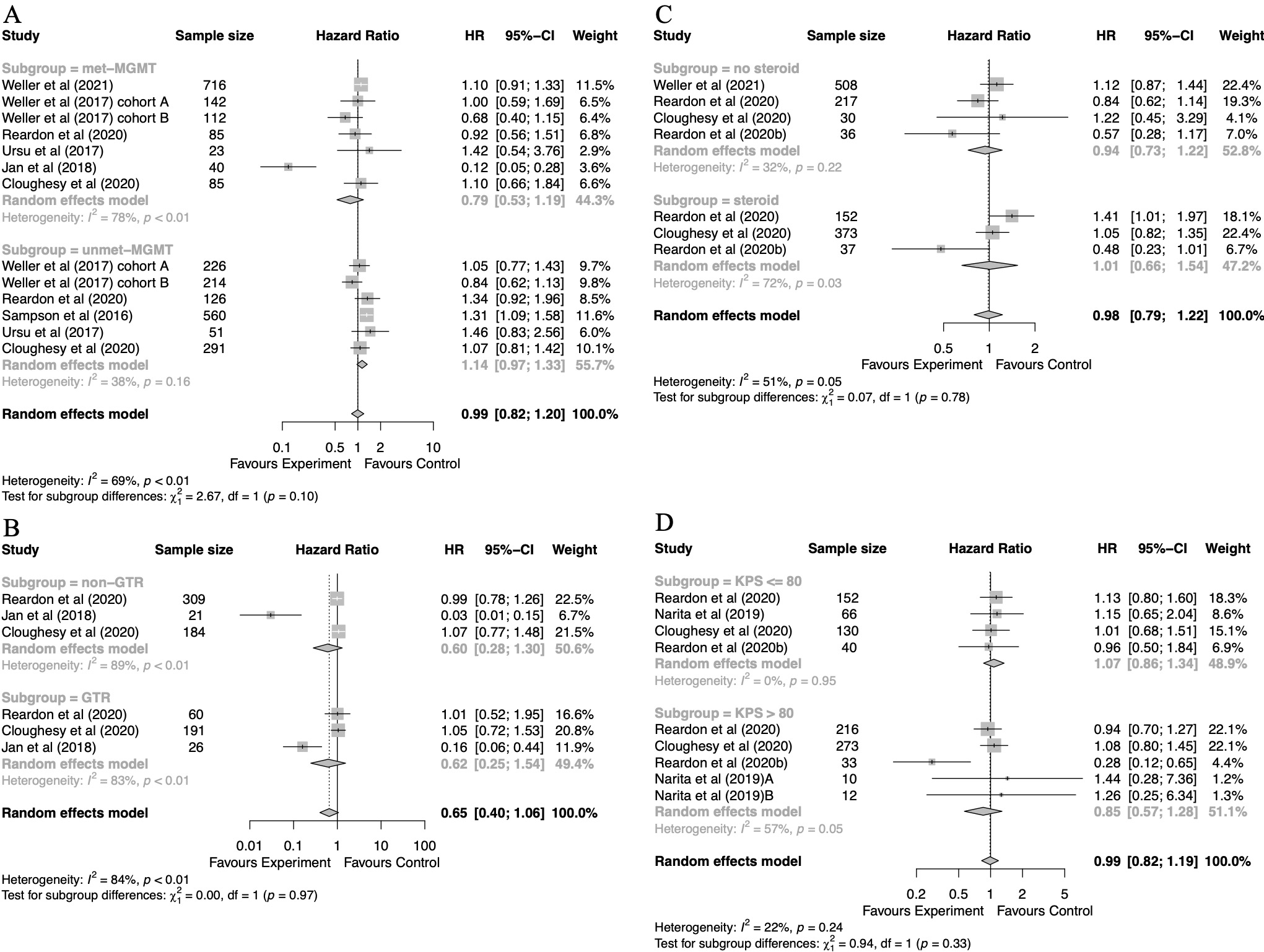

Supplement: Supplementary file 1 — Figure S1 [file CNS-28-1476-s006.jpg]

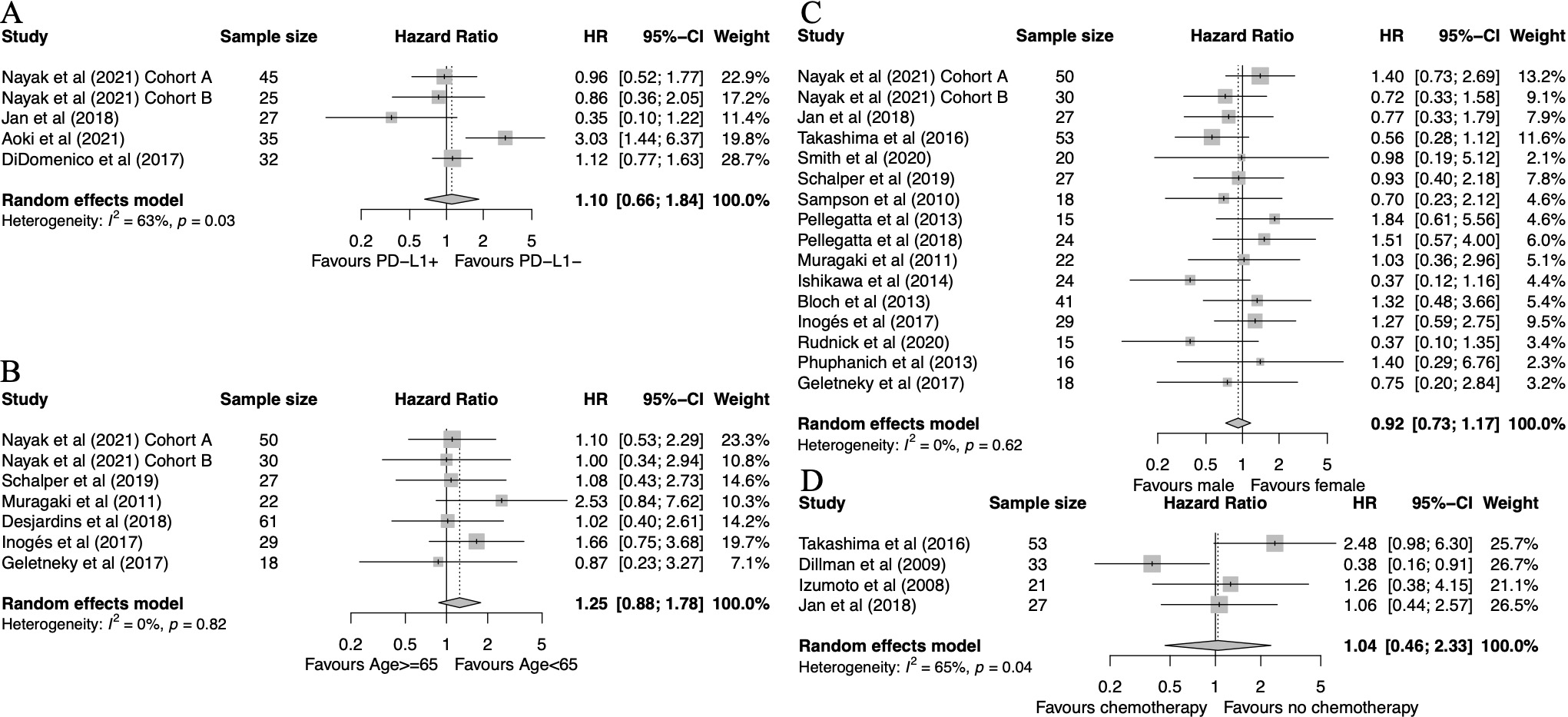

Supplement: Supplementary file 2 — Figure S2 [file CNS-28-1476-s004.jpg]

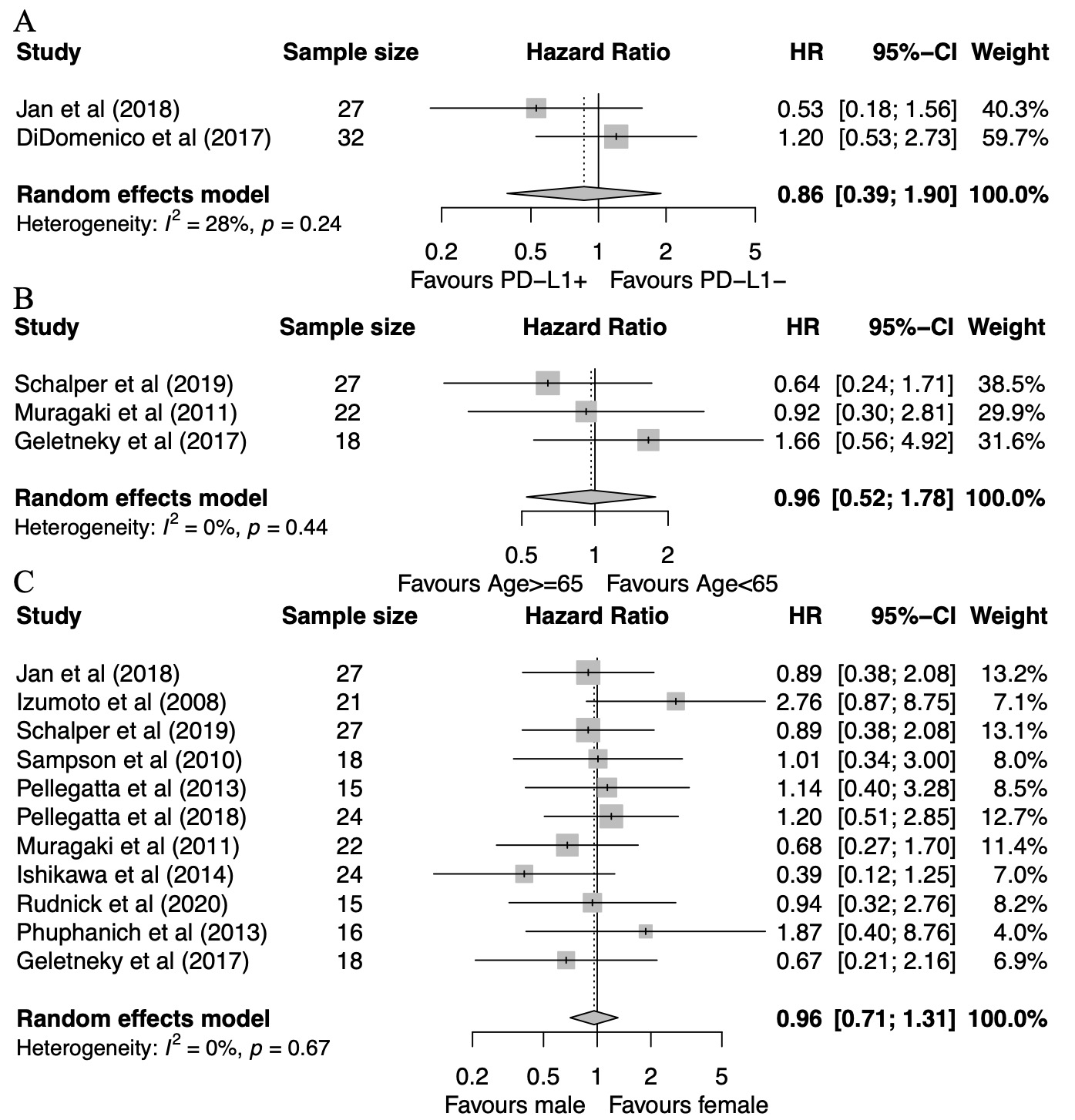

Supplement: Supplementary file 3 — Figure S3 [file CNS-28-1476-s002.jpg]

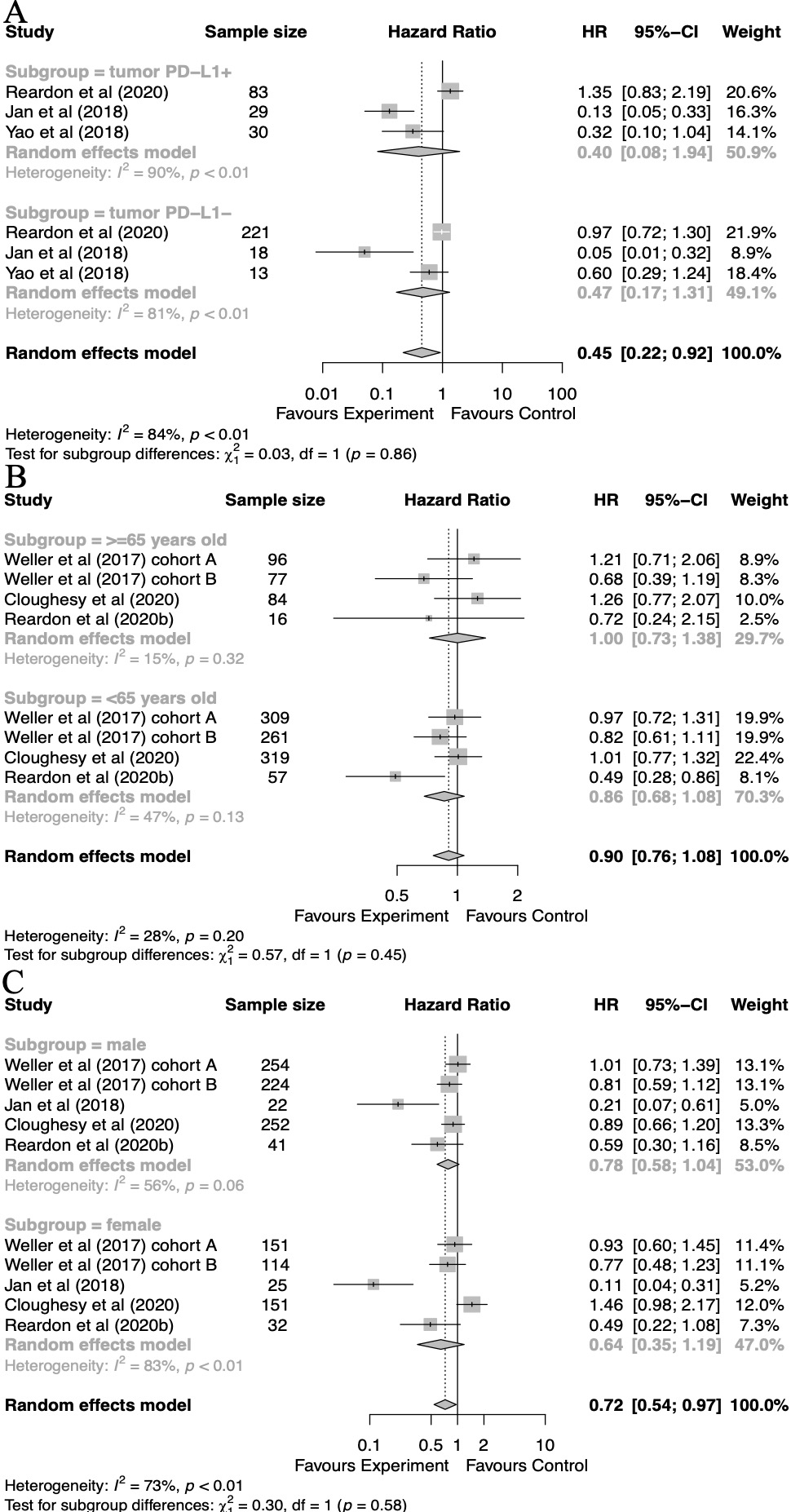

Supplement: Supplementary file 4 — Figure S4 [file CNS-28-1476-s008.jpg]

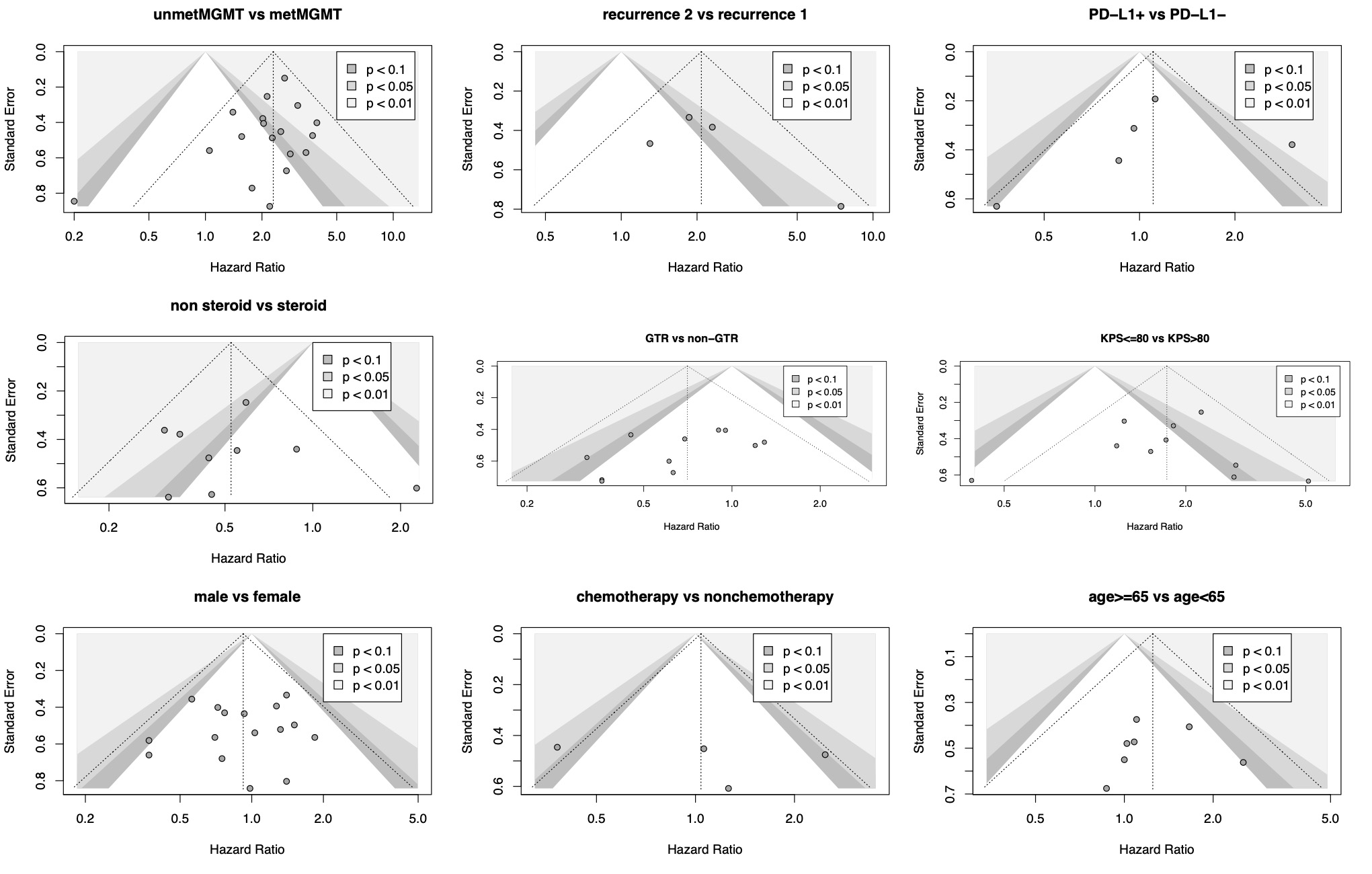

Supplement: Supplementary file 5 — Figure S5 [file CNS-28-1476-s003.jpg]

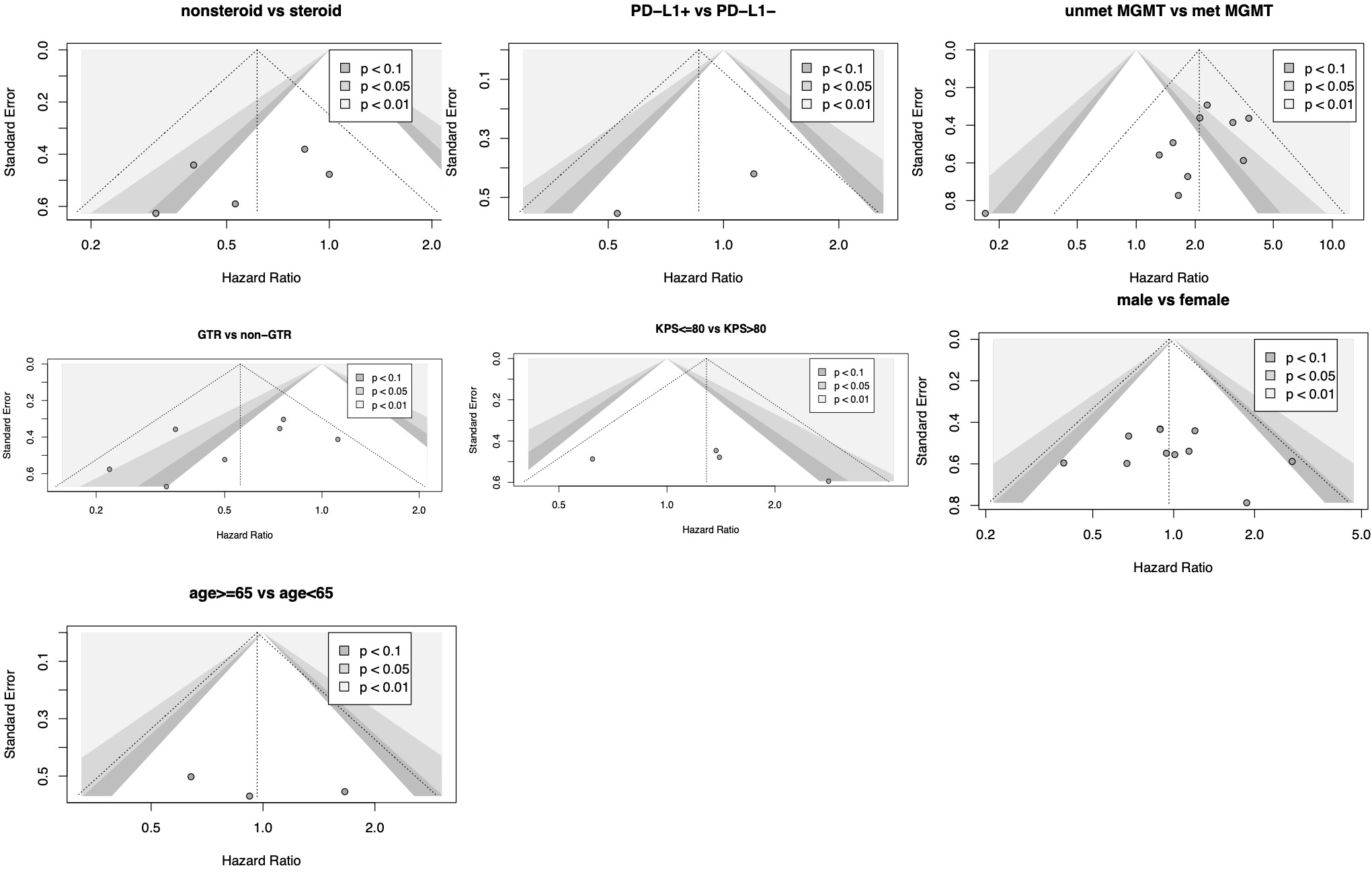

Supplement: Supplementary file 6 — Figure S6 [file CNS-28-1476-s001.jpg]

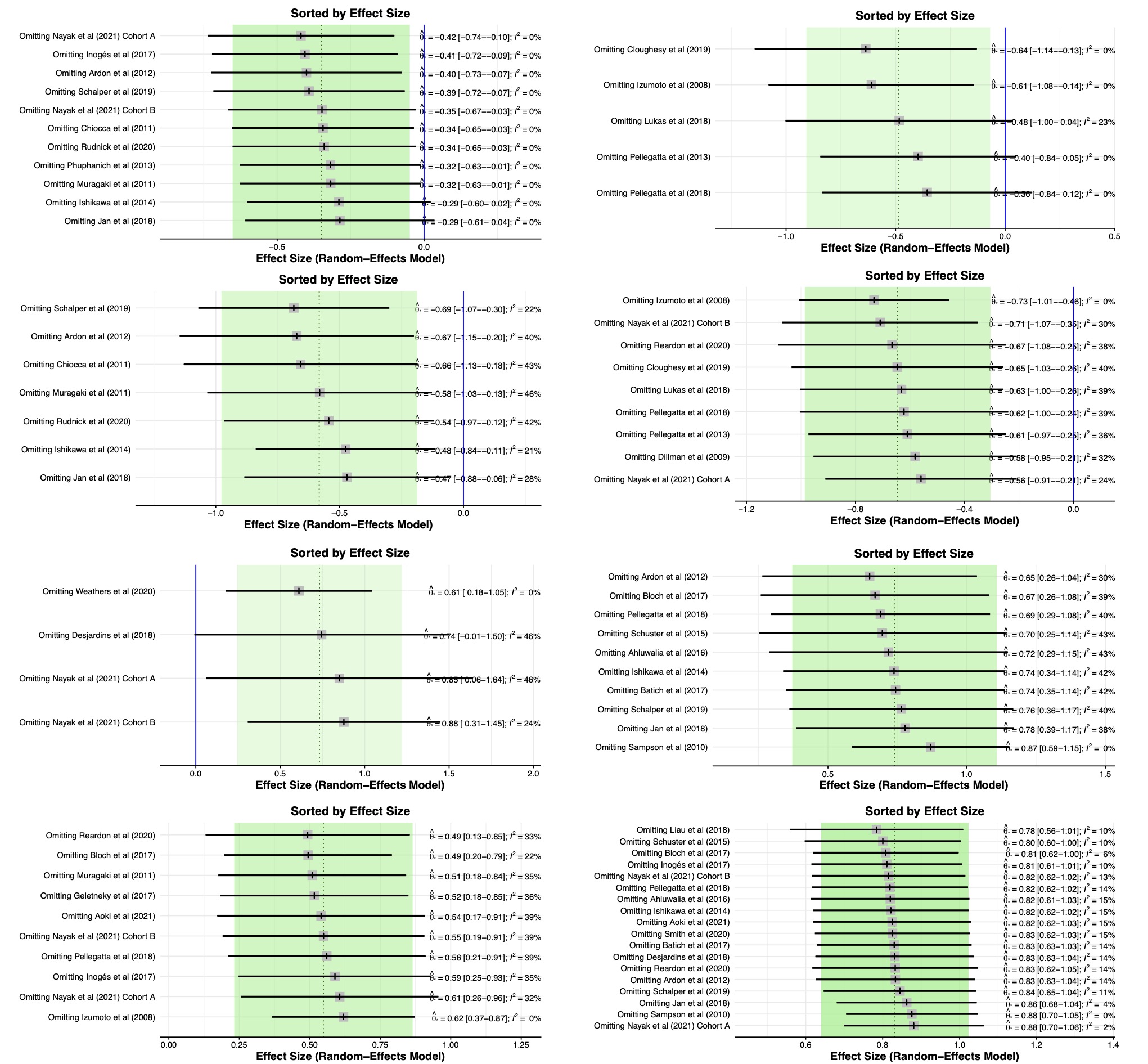

Supplement: Supplementary file 7 — Figure S7 [file CNS-28-1476-s007.jpg]
