# Supplementary material for: Impact of molecular and clinical variables on survival outcome with immunotherapy for glioblastoma patients: A systematic review and meta‐analysis
Source: CNS Neurosci Ther. 2022 Jul 13;28(10):1476–91. doi: 10.1111/cns.13915 (PMC9437230; doi:10.1111/cns.13915)
Supplement: Supplementary file 8 — Table S1 [file CNS-28-1476-s009.docx]

Table S1 The risk of bias of randomized trials by RoB2

| Study | Domain 1 | Domain 2 | Domain 3 | Domain 4 | Domain 5 | Overall |
| --- | --- | --- | --- | --- | --- | --- |
| Reardon et al (2020) | Low risk | Some concerns | Low risk | Low risk | Low risk | Some concerns |
| Liau et al (2018) | Low risk | Low risk | Some concerns | Low risk | Low risk | Some concerns |
| Nayak et al (2021) | Some concerns | Low risk | Low risk | Low risk | Low risk | Some concerns |
| Jan et al (2018) | Low risk | Some concerns | Low risk | Low risk | Low risk | Some concerns |
| Cloughesy et al (2019) | Some concerns | Some concerns | Low risk | Low risk | Low risk | Some concerns |
| Cloughesy et al (2020) | Low risk | Some concerns | Low risk | Low risk | Low risk | Some concerns |
| Weller et al (2021) | Low risk | Low risk | Low risk | Low risk | Low risk | Low risk |
| Weller et al (2017) | Low risk | Low risk | Low risk | Low risk | Low risk | Low risk |
| Ursu et al (2017) | Low risk | Some concerns | Low risk | Low risk | Low risk | Some concerns |
| Sampson et al (2016) | Low risk | Some concerns | Low risk | Low risk | Low risk | Some concerns |
| Narita et al (2019) | Low risk | Low risk | Low risk | Low risk | Low risk | Low risk |
| Reardon et al (2020b) | Low risk | Low risk | Low risk | Low risk | Low risk | Low risk |
| Yao et al (2018) | Low risk | Some concerns | Low risk | Low risk | Low risk | Some concerns |

Scoring items: Domain 1: Bias arising from the randomisation process; Domain 2: Bias due to deviations from intended interventions; Domain 3: Bias due to missing outcome data; Domain 4: Bias in measurement of the outcome; Domain 5: Bias in selection of the reported results
