# Supplementary material for: Impact of molecular and clinical variables on survival outcome with immunotherapy for glioblastoma patients: A systematic review and meta‐analysis
Source: CNS Neurosci Ther. 2022 Jul 13;28(10):1476–91. doi: 10.1111/cns.13915 (PMC9437230; doi:10.1111/cns.13915)
Supplement: Supplementary file 9 — Table S2 [file CNS-28-1476-s005.docx]

Table S2 The methodological quality assessment of non-randomized studies by MINORS

| Study | ① | ② | ③ | ④ | ⑤ | ⑥ | ⑦ | ⑧ | Total scores |
| --- | --- | --- | --- | --- | --- | --- | --- | --- | --- |
| Inogés et al (2017) | 2 | 2 | 2 | 2 | 1 | 2 | 2 | 2 | 15 |
| Ardon et al (2012) | 2 | 2 | 2 | 2 | 1 | 2 | 1 | 1 | 13 |
| Bloch et al (2017) | 2 | 2 | 2 | 2 | 1 | 1 | 2 | 2 | 14 |
| Ahluwalia et al (2016) | 2 | 2 | 2 | 2 | 1 | 1 | 1 | 2 | 13 |
| Smith et al (2020) | 2 | 2 | 2 | 2 | 1 | 1 | 1 | 2 | 13 |
| Batich et al (2017) | 2 | 2 | 2 | 2 | 1 | 1 | 1 | 2 | 13 |
| Pellegatta et al (2018) | 2 | 2 | 2 | 2 | 1 | 2 | 2 | 2 | 15 |
| Ishikawa et al (2014) | 2 | 2 | 2 | 2 | 2 | 1 | 2 | 2 | 15 |
| Schuster et al (2015) | 2 | 2 | 2 | 2 | 1 | 2 | 1 | 2 | 14 |
| Schalper et al (2019) | 2 | 2 | 2 | 2 | 1 | 2 | 2 | 2 | 15 |
| Sampson et al (2010) | 2 | 2 | 2 | 2 | 1 | 2 | 1 | 2 | 14 |
| Aoki et al (2021) | 2 | 2 | 2 | 2 | 1 | 1 | 1 | 2 | 13 |
| Desjardins et al (2018) | 2 | 2 | 2 | 2 | 1 | 2 | 1 | 2 | 14 |
| Izumoto et al (2008) | 2 | 2 | 2 | 2 | 1 | 1 | 1 | 0 | 11 |
| Pellegatta et al (2013) | 2 | 2 | 2 | 2 | 1 | 2 | 2 | 2 | 15 |
| Lukas et al (2018) | 2 | 2 | 2 | 2 | 1 | 2 | 2 | 1 | 14 |
| Dillman et al (2009) | 2 | 2 | 2 | 2 | 1 | 1 | 1 | 1 | 12 |
| Muragaki et al (2011) | 2 | 2 | 2 | 2 | 1 | 1 | 2 | 2 | 14 |
| Geletneky et al (2017) | 2 | 2 | 2 | 2 | 1 | 1 | 1 | 0 | 11 |
| Takashima et al (2016) | 2 | 2 | 2 | 2 | 1 | 1 | 1 | 1 | 12 |
| Bloch et al (2013) | 2 | 2 | 2 | 2 | 1 | 2 | 2 | 2 | 15 |
| Rudnick et al (2020) | 2 | 2 | 2 | 2 | 1 | 2 | 1 | 2 | 14 |
| Phuphanich et al (2013) | 2 | 2 | 2 | 2 | 1 | 1 | 2 | 2 | 14 |
| Chiocca et al (2011) | 2 | 2 | 2 | 2 | 1 | 2 | 2 | 1 | 14 |
| DiDomenico et al (2017) | 2 | 2 | 2 | 2 | 1 | 1 | 2 | 2 | 14 |
| Weathers et al (2020) | 2 | 2 | 2 | 2 | 1 | 2 | 2 | 2 | 15 |

Scoring items: ① A clearly stated aim: ② Inclusion of consecutive patients; ③ Prospective collection of data; ④ Endpoints appropriate to the aim of the study; ⑤ Unbiased assessment of the study endpoint; ⑥ Follow-up period appropriate to the aim of the study; ⑦ Loss to follow up less than 5%; ⑧ Prospective calculation of the study
